# Gas-controlled capillary spinner for time-resolved powder X-ray diffraction under dynamic conditions

**DOI:** 10.1107/S160057672600645X

**Published:** 2026-07-29

**Authors:** Shogo Kawaguchi, Michitaka Takemoto, Shintaro Kobayashi, Yoshiki Kubota

**Affiliations:** a Japan Synchrotron Radiation Research Institute (JASRI) 1-1-1 Kouto Sayo-cho, Sayo-gun Hyogo679-5198 Japan; b Osaka Metropolitan University, 3-3-138 Sugimoto, Sumiyoshi-ku, Osaka558-8585, Japan; HPSTAR and Harbin Institute of Technology, People’s Republic of China

**Keywords:** time-resolved powder diffraction, *in situ*X-ray diffraction, sample environments, gas-controlled capillary spinners, dynamic gas conditions

## Abstract

A gas-controlled capillary spinner was developed for time-resolved powder X-ray diffraction measurements of crystalline materials under controlled atmospheres. This system enables continuous sample rotation with simultaneous gas control by integrating a gas-tight capillary cell, a magnetic fluid rotary feedthrough and a fast-response diaphragm valve, allowing diffraction measurements to be performed in rapidly changing environments. The system performance was evaluated using a standard Si powder sample, revealing that high-speed rotation significantly improves the uniformity of the diffraction peak intensity, with relative fluctuations reduced to <1% at speeds of ∼200 r min^−1^ even with millisecond-scale measurements. The applicability of the system was demonstrated by time-resolved diffraction measurements of a metal–organic framework during gas adsorption, in which structural changes were successfully tracked and quantitatively analyzed with a time resolution of 50 ms. These findings indicate that the developed system provides a reliable approach for *in situ* and *operando* diffraction studies under dynamic gas conditions.

## Abstract 2

The newly developed gas-controlled capillary spinner enables continuous high-speed sample rotation during *in situ* powder X-ray diffraction under dynamic gas conditions. By improving the uniformity of the diffraction intensity, this system enables reliable millisecond time-resolved structural analysis for *operando* studies.

## 1. Introduction

Gas adsorption, separation and catalytic processes occurring in functional materials – such as metal–organic frameworks, nanoporous materials and catalysts – have attracted significant attention in recent years owing to their importance for energy, environmental and industrial applications. Understanding the structural responses of these materials under working conditions is therefore essential for elucidating their functional mechanisms. *In situ* powder X-ray diffraction (XRD) measurements under controlled gas atmospheres provide a powerful approach for probing such structural changes during gas adsorption and desorption processes (Horike *et al.*, 2009 ▸; Schneemann *et al.*, 2014 ▸). These measurements enable precise control of the gas environments and reliable structural characterization under dynamic conditions. Recent advances in X-ray sources and 2D hybrid detectors have enabled time-resolved diffraction measurements with one-second and sub-second time resolution during continuous processes, providing new opportunities for investigating dynamic structural changes (Garai *et al.*, 2021 ▸; Ashitani *et al.*, 2023 ▸; Sakanaka *et al.*, 2023 ▸). Consequently, there is an increasing demand for experimental setups capable of reliable diffraction measurements under such rapid and dynamically changing conditions.

Continuous capillary rotation is important for *in situ* powder XRD measurements under controlled gas atmospheres, as it improves particle statistics and enables uniform diffraction intensities while maintaining gas-tight conditions. Traditionally, several approaches have been employed to combine gas control and capillary-based measurements in transmission powder XRD measurements using synchrotron radiation. One approach involves fixing the capillary to a goniometer head using adhesive materials, allowing static measurements under controlled atmospheres (Kubota *et al.*, 2007 ▸; Jensen *et al.*, 2010 ▸; Hill, 2013 ▸). In such configurations, continuous rotation can only be achieved after disconnecting the gas lines. As a result, gas exchange during continuous rotation is difficult. Another approach employs specially designed gas cells that allow oscillation of the capillary while maintaining gas connections. Various types of cells have been developed to accommodate different experimental requirements, including gas–vapor adsorption studies, high-pressure experiments and continuous gas flow measurements (Kawaguchi *et al.*, 2020 ▸; Fraga *et al.*, 2019 ▸; Scarlett *et al.*, 2017 ▸). Although these methods improve particle statistics compared with static measurements, the angular motion remains limited owing to the constraints of the gas lines. Consequently, it is difficult to obtain uniform diffraction intensities from crystalline materials in fast time-resolved measurements with short exposure times.

To address these limitations, we have developed a gas-controlled capillary spinner system for performing *in situ* powder XRD measurements at beamlines BL02B2 and BL13XU, SPring-8, Japan. This system enables the continuous high-speed rotation of a glass capillary sample while allowing control of the gas atmosphere during rotation, overcoming a key limitation of conventional gas-cell setups. Additionally, high-speed rotation provides effective averaging of the diffraction intensity, enabling uniform diffraction data to be obtained even in fast time-resolved measurements. The system also allows the use of standard glass capillaries, facilitating sample exchange and practical operation. Flexible and dynamic control of the gas environments can be achieved by integrating a spinner with a remote gas-handling system. In the current study, the performance of the developed system is quantitatively evaluated in terms of diffraction-intensity uniformity as a function of rotation speed. Its applicability to fast time-resolved measurements under controlled gas atmospheres is also demonstrated.

## 2. Development of gas-controlled spinner

**Figure 1:**
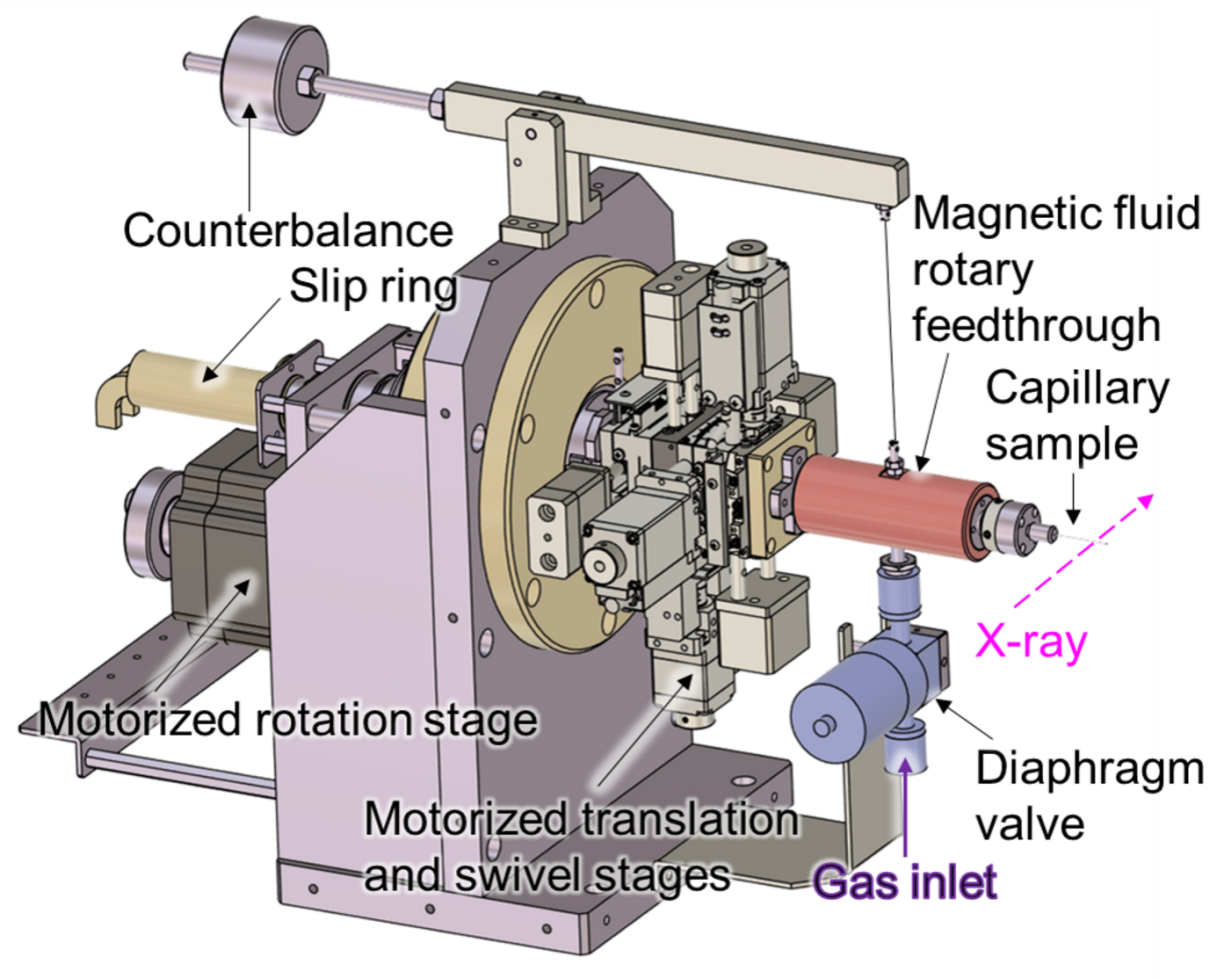
Schematic representation of the gas-controlled capillary spinner developed in this study.

This section describes the design and components of the newly developed gas-controlled capillary spinner for *in situ* powder XRD measurements under a controlled gas atmosphere. As shown in Fig. 1 ▸, the spinner consists of several key components: a capillary cell to support the glass capillary, a magnetic fluid rotary feedthrough, a fast-response diaphragm valve, two translation stages and two swivel stages for alignment, a motorized rotation stage for continuous spinning of the capillary, a slip ring for continuous rotation of electrical connections, and a counterbalance to stabilize the sample during high-speed operation.

The capillary cell was designed to accommodate a 0.5 mm diameter glass capillary, which is held using two O-rings and a polyether ether ketone tube, without the requirement for adhesives. The system can be adapted to other capillary diameters by simply exchanging internal components. Notably, the standard glass capillaries used in conventional XRD experiments can be directly applied without any modification. This design ensures both gas-tight sealing and facile sample exchange. A custom-made corrosion-resistant magnetic fluid rotary feedthrough was used in this study. The feedthrough is capable of rotation up to 1000 r min^−1^ while maintaining a gas-tight seal, and the system has been successfully operated with gases including N_2_, O_2_, Ar, CO_2_, H_2_, CH_4_ and C_2_H_2_. The rotation motor is equipped with a water-cooling system to reduce heat generation during operation. The counterbalance and the secure attachment of the gas tubing between the diaphragm valve of the spinner and the gas-handling system compensate for mechanical loads that could otherwise induce capillary eccentricity. Visual observation using the image-recognition camera showed that improper balancing of the tubing could result in capillary eccentricity exceeding ∼100 µm. Particular attention was therefore paid to the secure attachment of the gas tubing and the adjustment of the counterbalance. Operation in this study was limited to 600 r min^−1^ because prolonged operation at higher rotational speeds may increase heat generation in the motor and mechanical vibration of the spinner assembly, potentially affecting rotational stability. High-speed rotation is primarily intended for short-duration high-time-resolution measurements, typically lasting from several tens of seconds to a few minutes, thereby limiting heat buildup in both the motor and the rotary feedthrough. The gas-tight performance of the system was confirmed using a helium leak detector (HELIOT series, ULVAC). The capillary assembly was evacuated and tested by the helium spray method, showing a leakage rate below 1 × 10^−11^ Pa m^3^ s^−1^. The diaphragm valve, which is controlled by a remote gas-handling system developed at the beamline (Kawaguchi *et al.*, 2020 ▸), allows rapid gas switching by supplying compressed air, thereby enabling a fast response during time-resolved measurements. Translation and swivel stages are used for precise alignment of the capillary with respect to the incident X-ray beam. Additionally, automatic sample centering can be achieved using the image-recognition system. Using this configuration, stable high-speed rotation up to 600 r min^−1^ is achieved, while the typical operating speed is ∼200 r min^−1^. Furthermore, by integrating the spinner with the remote gas-handling system, the gas pressure can be controlled in the range of 1 Pa to 130 kPa. The system can be connected to a turbomolecular pump, enabling operation under high-vacuum conditions.

## 3. System performance and applicability

**Figure 2:**
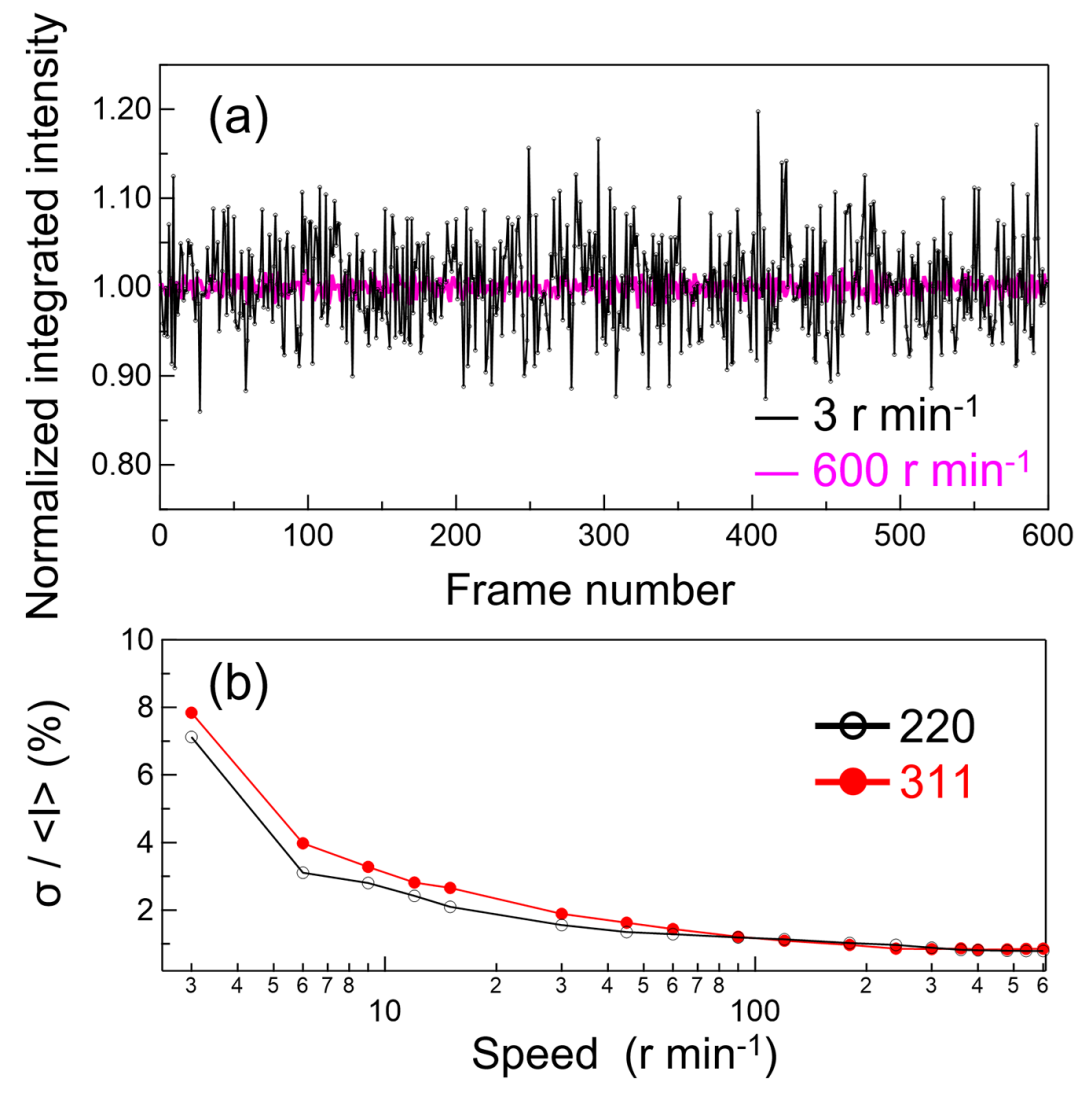
(*a*) Frame-to-frame variation in the normalized integrated intensity of the 220 reflection of Si powder at rotation speeds of 3 and 600 r min^−1^. The horizontal axis represents the frame number, with an exposure time of 10 ms per frame. (*b*) Relative fluctuation of the integrated intensities (σ/〈*I*〉) for the 220 and 311 reflections as a function of rotation speed. The fluctuation decreases with increasing rotation speed and reaches <1% above ∼200 r min^−1^, indicating improved intensity uniformity.

To evaluate the effect of rotation speed on diffraction-intensity uniformity, continuous measurements were per­formed using a standard Si powder sample (NIST SRM 640c) loaded in a 0.5 mm glass capillary. The experiments were conducted using a high-resolution powder diffractometer (BL13XU, SPring-8) with an X-ray energy of 35 keV. Diffraction data were collected using six sets of 2D CdTe detectors (LAMBDA 750k) in the single-step mode (Kawaguchi *et al.*, 2024 ▸). Measurements were performed with an exposure time of 10 ms over 600 consecutive frames while varying the rotation speed. Fig. 2 ▸(*a*) shows the normalized integrated intensity of the 220 reflection as a function of the frame number at rotation speeds of 3 and 600 r min^−1^. The intensity was normalized to the average intensity over 600 frames. At 3 r min^−1^, significant frame-to-frame fluctuations were observed in the diffraction intensity, indicating insufficient averaging of the crystallite orientations during measurement. In contrast, at 600 r min^−1^, the intensity became substantially more stable, demonstrating improved intensity uniformity over a short exposure time. In oscillation measurements using gas cells performed at BL02B2 (Kawaguchi *et al.*, 2017 ▸) and BL13XU, the effective rotation speed was typically below 1 r min^−1^, corresponding to angular scan conditions of ∼2–10 s per degree (∼0.1 r min^−1^). Under such conditions, the angular motion during a 10 ms exposure is extremely limited, rendering it difficult to obtain a uniform diffraction intensity. These results indicate that high-speed rotation is essential for obtaining a stable diffraction intensity in fast time-resolved measurements.

Fig. 2 ▸(*b*) shows the relative fluctuation of the integrated intensities of the 220 and 311 reflections of Si as a function of rotation speed. The fluctuation is defined as σ/〈*I*〉, where σ and 〈*I*〉 denote the standard deviation and mean intensity, respectively, as calculated from 600 consecutive frames with an exposure time of 10 ms. The integrated intensities were obtained by peak integration of each frame after background subtraction. The relative fluctuation decreased monotonically with increasing rotation speed, reflecting the improved averaging of the crystallite orientations during the measurement. Additionally, the fluctuation was significantly reduced to <1% at 200 r min^−1^, above which further improvement became limited. This behavior can be interpreted as the condition under which a sufficient number of crystallites pass through the X-ray beam within a single exposure time. Thus, a rotation speed of ∼200 r min^−1^ achieved a stable diffraction intensity under the present experimental conditions. A similar trend was observed for other reflections, where the relative fluctuation decreased with increasing rotation speed, although the absolute values depend on the reflection intensity and counting statistics. No noticeable changes were observed in the peak positions or profiles, suggesting that capillary eccentricity during high-speed rotation is negligible. Specifically, at a rotation speed of 600 r min^−1^, the standard deviations of the peak position and the full width at half-maximum for the 220 reflection over 600 consecutive frames were 0.0002° and 0.0004°, respectively. These values are significantly smaller than the angular step size of the detector (0.005°), indicating that high-speed rotation has a negligible effect on peak shape and position. Although the exact values depend on the sample properties – such as crystallinity, particle size and measurement conditions – these results provide a practical guideline for rotation-speed selection in fast time-resolved measurements.

**Figure 3:**
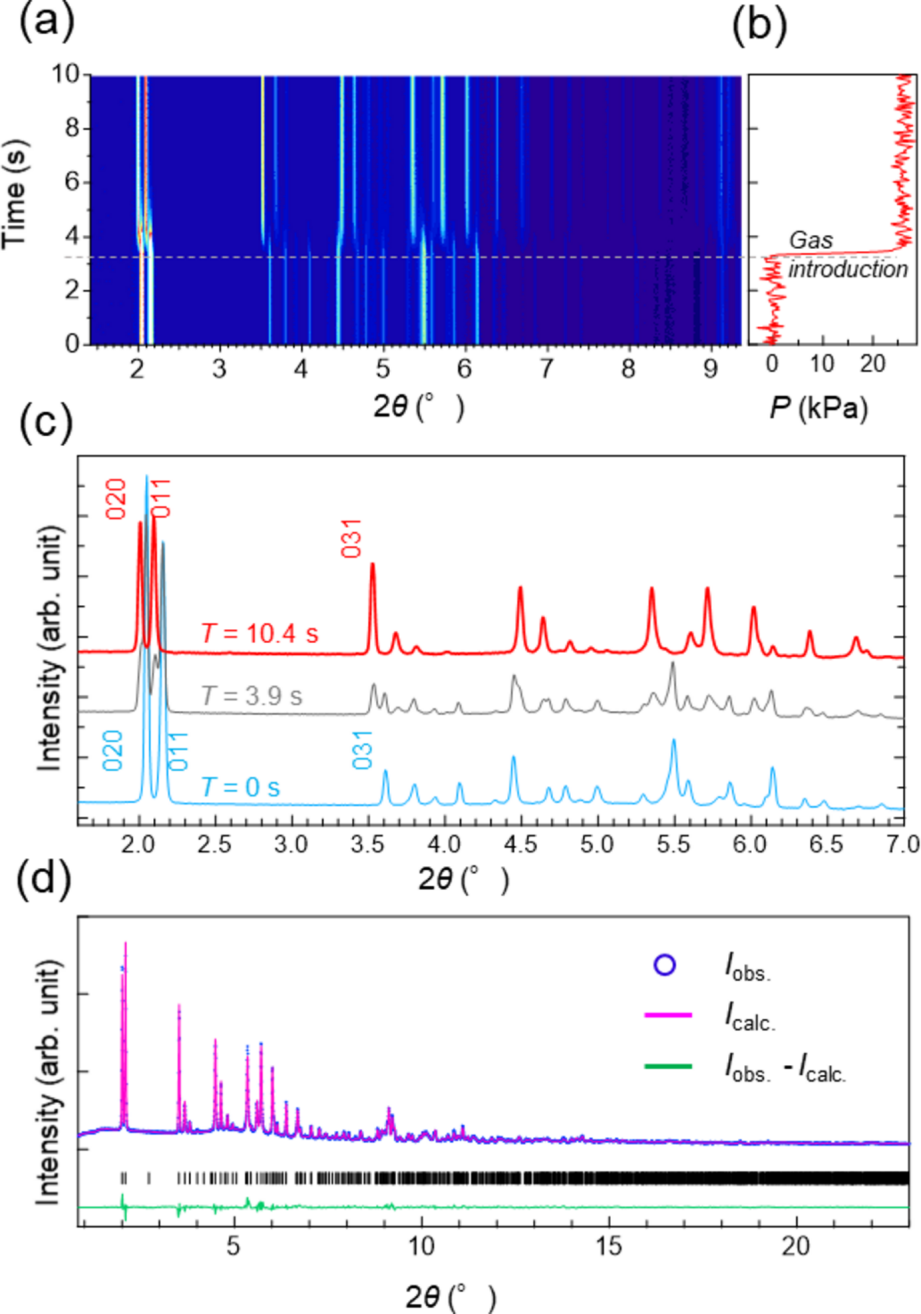
(*a*) Time-resolved powder diffraction intensity map of the CPL-1 sample at 100 K using an X-ray energy of 35 keV. (*b*) Time dependence of the Ar gas pressure. (*c*) Representative diffraction patterns recorded before gas introduction (*T* = 0 s), shortly after Ar introduction (*T* = 3.9 s) and after Ar adsorption (*T* = 10.4 s). (*d*) Rietveld refinement results for the diffraction pattern recorded with a 50 ms exposure time after Ar gas adsorption. The observed and calculated profiles, the difference plot, and the Bragg positions are shown.

Finally, the performance of the developed system was evaluated under dynamic gas conditions by performing time-resolved diffraction measurements on a metal–organic framework (CPL-1) during gas adsorption. This material is a nanoporous Cu coordination polymer with the formula [Cu_2_(pzdc)_2_(pyz)]_*n*_ (pzdc = pyrazine-2,3-di­carboxyl­ate; pyz = pyrazine) and is known to exhibit typical physisorption behavior (Type I isotherm) in the adsorption of various gases (Kitaura *et al.*, 2002 ▸, 2005 ▸). The sample was cooled to 100 K using a nitro­gen gas blower, and diffraction data were collected with continuous acquisition at 50 ms per frame. Prior to gas introduction, the sample capillary was evacuated. To facilitate rapid and homogeneous gas penetration, the capillary length was kept short (∼20 mm) and the powder sample was loaded over a length of only ∼1 mm. The gas-pressure control system was synchronized with the detector, and Ar gas was introduced at 26 kPa approximately 3 s after the start of the measurement. Fig. 3 ▸(*a*) shows the time evolution of the diffraction patterns during gas introduction, presented as a color map. A pronounced change in the diffraction pattern can be observed immediately after the introduction of Ar gas (∼3 s after the start of the measurement), accompanied by changes in the peak intensities and slight shifts in the peak positions. These changes indicate a structural transition from the desorbed phase to the adsorbed phase. Notably, the timing of this transition coincided with an increase in the gas pressure, as shown in Fig. 3 ▸(*b*), confirming that the observed structural changes were induced by gas adsorption. Additionally, the continuous evolution of the diffraction intensity demonstrates that the structural response of the framework can be tracked in real time under dynamic gas conditions. Representative diffraction patterns recorded before gas introduction (*T* = 0 s), shortly after Ar introduction (*T* = 3.9 s) and after Ar adsorption (*T* = 10.4 s) are compared in Fig. 3 ▸(*c*). Distinct changes in both peak positions and relative intensities are observed, particularly for the 020, 011 and 031 reflections, indicating the structural transformation associated with Ar adsorption. Fig. 3 ▸(*d*) shows the Rietveld refinement results for the powder XRD pattern recorded at an exposure time of 50 ms after Ar gas adsorption. The refinement was performed using a structural model of the adsorbed phase previously reported from static measurements (Kitaura *et al.*, 2005 ▸), without refining atomic positional parameters. A satisfactory fit was obtained, with weighted-profile *R* factor (*R*_wp_) and Bragg *R* factor (*R*_B_) values of ∼4% and 5%, respectively, indicating good agreement between the observed and calculated diffraction patterns. Successful refinement of the diffraction pattern acquired within 50 ms indicates that quantitative structural analysis is feasible even under such rapid measurement conditions during gas adsorption.

## 4. Conclusions

This study demonstrates the performance of a gas-controlled capillary spinner for time-resolved powder X-ray diffraction measurements performed under dynamic conditions. This system enables continuous sample rotation with simultaneous gas control, allowing stable diffraction measurements in rapidly changing environments. The capillary cell supports rotation speeds of up to 600 r min^−1^, and high-speed rotation significantly improves the diffraction-intensity uniformity, enabling reliable structural analysis even with short acquisition times (*i.e.* tens of milliseconds). These results indicate that the developed system provides a practical approach for time-resolved diffraction studies in controlled gas environments. This method is expected to be particularly useful for experiments requiring fast data acquisition, such as those performed at advanced synchrotron sources, as well as for *operando* studies of transient processes including gas adsorption and catalytic reactions. The present system is primarily intended for dynamic gas-switching experiments under near-ambient-pressure conditions. Further expansion of the pressure range through improvements in the rotary sealing system is an important direction for future development.
